# Saul Wilson Syndrome: A Case Report With New Features in Saudi Arabia

**DOI:** 10.1002/ccr3.71834

**Published:** 2026-02-08

**Authors:** Saad A. Bin Owaimer, Fatimah H. Abusrair, May R. Mutlaq, Eissa A. Faqeih, Leen Abu‐Safieh

**Affiliations:** ^1^ Physician, General Pediatrics National Guard Hospital Riyadh Kingdom of Saudi Arabia; ^2^ Physician Dar Al‐Uloom University Riyadh Kingdom of Saudi Arabia; ^3^ Physician, Sulaiman Alrajhi University Al‐Qassim Kingdom of Saudi Arabia; ^4^ Consultant, Clinical and Metabolic Genetics King Fahad Medical City Riyadh Kingdom of Saudi Arabia; ^5^ Consultant, Clinical Research, Bioinformatics and Computational Biology Department King Fahad Medical City Riyadh Kingdom of Saudi Arabia

**Keywords:** case report, COG4, primordial dwarfism, Saul‐Wilson syndrome, skeletal dysplasia

## Abstract

Saul Wilson syndrome is an extremely rare genetic disorder caused by heterozygous de novo mutations in the *COG4* gene. We report the first case from Saudi Arabia with previously unreported facial dysmorphic features, expanding the known phenotypic spectrum and emphasizing the importance of recognizing phenotypic variability in rare disorders.

## 1 Introduction

Saul Wilson syndrome (SWS) is a rare condition of microcephalic primordial dwarfism. A heterozygous de novo missense mutation (p.Gly516Arg) in the *COG4* gene, which encodes one of eight subunits of the conserved oligomeric Golgi complex, has been identified as a leading cause of this condition [1, 2]. The Oligomeric Golgi complex is known to control the vesicular protein transportation between the Golgi apparatus and the endoplasmic reticulum and consist of two lobes with eight subunits; lobe A (COG1‐4), and lobe B (COG5‐8). SWS is characterized by growth retardation during the prenatal and postnatal periods with skeletal, ocular, hearing, and radiological manifestations [2, 3].

### 1.1 Dysmorphic Features and Skeletal Dysplastic Abnormalities

Affected patients were found to have a large anterior fontanelle, short stature, platyspondyly, dense hypoplasia, talipes clubfoot, and over tubulation of long bones. Additionally, patients usually have facial dysmorphisms including a temporary progeroid feature that appears during early infantile life and disappears a few months later, prominent scalp veins, and changing the shape of the forehead from round to taller and less rounded [3].

### 1.2 Growth and Development

Affected individuals had impaired growth, with a dramatic decline in growth in the first months of life. Speech and motor skills were also delayed in those patients, while cognitive function was reserved [4].

### 1.3 Ocular and Hearing Manifestations

The previously reported cases of SWS experienced blue sclera during early infancy, lamellar cataracts, nystagmus, pigmentary retinopathy with a rod‐cone dystrophy. The most common complication of SWS is hearing loss, which could be conductive, sensorineural, or mixed and requires a hearing aid [3].

### 1.4 Radiological Changes

These include overtubulation of long bones, odontoid process hypoplasia, platyspondyly, megaepiphyses, narrowed diaphysis and flaring of metaphysis. Flat vertebral bodies become taller with irregular endplates with aging. Patients were prone to fractures with minimal trauma, along with early manifestations of premature degenerative joint diseases [5].

### 1.5 Laboratory Abnormalities

Asymptomatic increase in the level of transaminase with AST more than ALT and intermittent neutropenia were observed, increasing their susceptibility to recurrent infections [5].

The diagnosis of SWS in patients with clear distinctive features is established with a gene‐targeted test, while in patients who present with unfamiliar manifestations, comprehensive genome testing is carried out [3].

Herein, we are reporting the first case of SWS in the Kingdom of Saudi Arabia with more distinctive clinical features that were not previously reported.

## 2 Case History\Examination

A 4‐year‐old girl, the firstborn child of healthy first‐degree relatives, was referred to our hospital for evaluation of global developmental delay and a history of recurrent infections since birth.

During the prenatal and perinatal periods, the pregnancy was complicated by oligohydramnios and intrauterine growth restriction. She was delivered by cesarean section at 29 weeks of gestation with an extremely low birth weight of 950 g. She was admitted to the neonatal intensive care unit and discharged at 3 months of age.

During infancy and early childhood, she had multiple hospital admissions due to recurrent infections, including gastroenteritis, otitis media, and sepsis. She demonstrated failure to thrive, with growth parameters persistently below the 3rd percentile. The patient also exhibited significant language delay; at 3 years of age, she was able to produce only a few unintelligible words despite receiving speech therapy. Her hearing history involved fluctuating conductive hearing loss. Initial testing at 7 months of age showed bilateral middle‐ear effusion with normal hearing thresholds. At 2 years of age, detailed audiological testing revealed mild left‐sided conductive hearing loss with persistent middle‐ear effusion. Crucially, when tested again at 2 years and 8 months, during the period when her speech delay was most severe, the child showed hearing responses within normal limits. This suggests that the restricted speech development at this stage could not be caused solely by impaired hearing. Subsequently, at 4 years of age, her hearing status deteriorated again, with bilateral conductive hearing loss and persistent middle‐ear dysfunction. Bilateral ventilation tube insertion was performed.

Physical examination revealed dysmorphic features, including a triangular face, plagiocephaly, hypertelorism, low‐set ears, a thin upper lip, a long philtrum, and talipes (clubfoot). The clubfoot had been surgically corrected in infancy. The remainder of the systemic examination was unremarkable.

## 3 Differential Diagnosis, Investigations and Treatment

**FIGURE 1:**
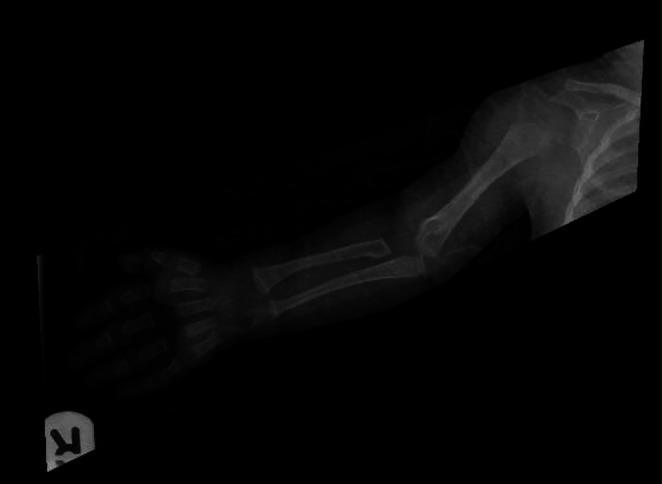
Skeletal survey at 8 months showing mild acromegaly and decreased bone mineralization with fraying, cupping, and widening of the metaphysis of the distal radius and ulna.

**FIGURE 2:**
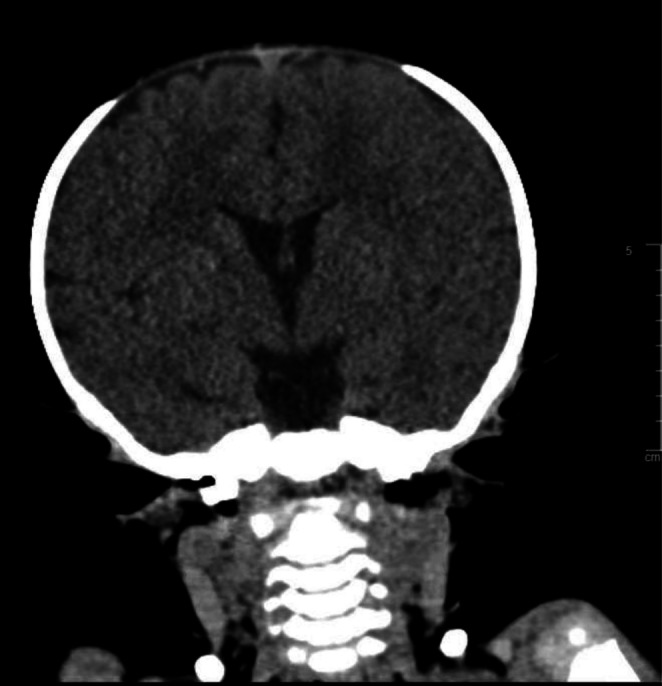
Non‐contrast CT brain at 15 months of age showing preserved brain parenchyma. There was widening of anterior fontanelle and sagittal sutures. The coronal sutures and particularly the temporoparietal sutures don't seem to be accurately open.

Radiological assessment at the age of 8 months, the patient did an ultrasound of the upper abdomen, which was normal. Her skeletal survey at that age interestingly showed mild acromegaly and diffusely decreased bone mineralization. There was fraying, cupping, and widening of the metaphysis of the distal radius and ulna bilaterally. Her 12‐pair ribs were identified bilaterally with no segmentation or formation vertebral anomaly. The sacral bone is well‐formed. The long bones of the upper and lower limbs are unremarkable. Hands and feet are unremarkable as well (as demonstrated in Figure 1). CT brain without contrast was done, at the age of 15 months, and showed preserved brain parenchyma. There was widening of anterior fontanelle and sagittal sutures. The coronal sutures and particularly the temporoparietal sutures don't seem to be accurately open (Figure 2). The patient's labs showed chronic moderately low neutrophil counts, monocytosis, and an elevated AST level without abnormalities in other liver function indices. Her vitamin D level was low. Lactate, ammonia, MS/MS, UOA, and parathyroid hormone were unremarkable. The bone marrow analysis was also normal. Patient karyotype analysis was normal; her cytogenetic/molecular FISH analysis and NGS hereditary/congenital neutropenia gene panel were negative. However, whole exome sequencing confirmed the diagnosis of SWS by identifying the pathogenic mutation p.Gly516Arg in the *COG4* gene.

## 4 Conclusion and Results (Outcome and Follow Up)

We reported a unique clinical finding for a patient with Saul Wilson syndrome confirmed by genetic testing. Reporting similar observations is necessary to collect a wide range of possible presentations and expand our understanding of this syndrome for early identification of patients in order to provide early medical intervention.

## 5 Discussion

We are reporting the first case of Saul Wilson syndrome in Saudi Arabia with distinctive features. The diagnosis was confirmed by the *COG4* mutation in a whole exome sequencing test along with the clinical findings. This is a very rare syndrome, with a total of 16 patients worldwide [3]. Our patient's clinical features and radiological findings were similar to previously reported features except for the triangular face, hypertelorism, and plagiocephaly. Furthermore, the patient's skeletal survey differed from the previously reported cases in which she had mild acromegaly and diffusely decreased bone mineralization. Intermittent neutropenia was noted in all reported cases [5]. However, our patient's neutropenic status was chronic, which explains her frequent infections. During medical follow‐up, the patient was started on Granulocyte colony‐stimulating factor (G‐CSF) twice a week, based on its effectiveness in significantly increasing neutrophil counts [5]. Nevertheless, our patient had a minor improvement in her absolute neutrophil count post‐G‐CSF administration.

SWS patients' need multidisciplinary support; ophthalmological, audiological, and motor assessments and complete blood counts are recommended annually. The treatment plan should be individualized according to the patient's manifestations, including skeletal surgery with osteotomy for skeletal dysplasia, and gastrostomy for optimizing the baby's nutrition and growth [3, 5].

## Author Contributions

**Eissa A. Faqeih:** project administration, resources, supervision, writing – original draft. **Fatimah H. Abusrair:** data curation, visualization, writing – original draft, writing – review and editing. **Saad A. Bin Owaimer:** data curation, visualization, writing – original draft, writing – review and editing. **Leen Abu‐Safieh:** supervision, validation, visualization, writing – review and editing. **May R. Mutlaq:** data curation, visualization, writing – original draft, writing – review and editing.

## Funding

The authors have nothing to report.

## Consent

Written informed consent was obtained from the patient's parents for publication of this case report and associated images.

## Conflicts of Interest

The authors declare no conflicts of interest.

## Data Availability

The data supporting the findings of this case report are available from the corresponding author upon reasonable request. However, they are not publicly available due to privacy and ethical restrictions.
